# How Far Can One Push the Noble Gases Towards Bonding?: A Personal Account

**DOI:** 10.3390/molecules24162933

**Published:** 2019-08-13

**Authors:** Ranajit Saha, Gourhari Jana, Sudip Pan, Gabriel Merino, Pratim Kumar Chattaraj

**Affiliations:** 1 Department of Chemistry and Centre for Theoretical Studies Indian Institute of Technology Kharagpur, Kharagpur 721302, India; 2 Institute of Advanced Synthesis, School of Chemistry and Molecular Engineering, Jiangsu National Synergetic Innovation Center for Advanced Materials, Nanjing Tech University, Nanjing 211816, China; 3 Departamento de Física Aplicada, Centro de Investigación y de Estudios Avanzados, Unidad Mérida. Km 6 Antigua Carretera a Progreso. Apdo. Postal 73, Cordemex, Mérida 97310, Yuc., Mexico; 4 Department of Chemistry, Indian Institute of Technology Bombay, Mumbai 400076, India

**Keywords:** noble gas, electron density, bonding, electron localization function, energy decomposition analysis

## Abstract

Noble gases (Ngs) are the least reactive elements in the periodic table towards chemical bond formation when compared with other elements because of their completely filled valence electronic configuration. Very often, extreme conditions like low temperatures, high pressures and very reactive reagents are required for them to form meaningful chemical bonds with other elements. In this personal account, we summarize our works to date on Ng complexes where we attempted to theoretically predict viable Ng complexes having strong bonding to synthesize them under close to ambient conditions. Our works cover three different types of Ng complexes, *viz.*, non-insertion of NgXY type, insertion of XNgY type and Ng encapsulated cage complexes where X and Y can represent any atom or group of atoms. While the first category of Ng complexes can be thermochemically stable at a certain temperature depending on the strength of the Ng-X bond, the latter two categories are kinetically stable, and therefore, their viability and the corresponding conditions depend on the size of the activation barrier associated with the release of Ng atom(s). Our major focus was devoted to understand the bonding situation in these complexes by employing the available state-of-the-art theoretic tools like natural bond orbital, electron density, and energy decomposition analyses in combination with the natural orbital for chemical valence theory. Intriguingly, these three types of complexes represent three different types of bonding scenarios. In NgXY, the strength of the donor-acceptor Ng→XY interaction depends on the polarizing power of binding the X center to draw the rather rigid electron density of Ng towards itself, and sometimes involvement of such orbitals becomes large enough, particularly for heavier Ng elements, to consider them as covalent bonds. On the other hand, in most of the XNgY cases, Ng forms an electron-shared covalent bond with X while interacting electrostatically with Y representing itself as [XNg]^+^Y^−^. Nevertheless, in some of the rare cases like NCNgNSi, both the C-Ng and Ng-N bonds can be represented as electron-shared covalent bonds. On the other hand, a cage host is an excellent moiety to examine the limits that can be pushed to attain bonding between two Ng atoms (even for He) at high pressure. The confinement effect by a small cage-like B_12_N_12_ can even induce some covalent interaction within two He atoms in the He_2_@B_12_N_12_ complex.

## 1. Introduction

**Figure 1:**
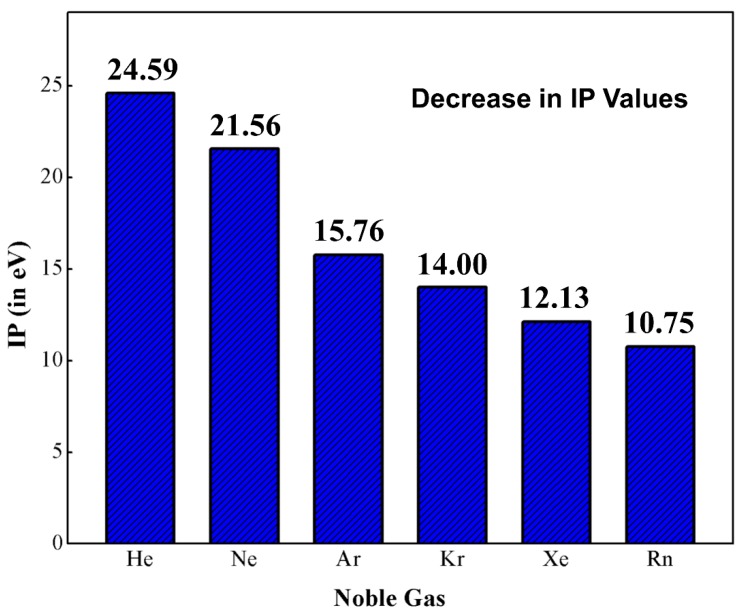
The plot of first ionization potential (IP; eV) against noble gas elements.

Helium (He) and argon (Ar) are the first noble gas (Ng) elements that found a place in the Mendeleev’s periodic table as group ‘0’ members [1]. The modern version of the periodic table has a total of seven Ng elements, *viz.*, helium (He), neon (Ne), argon (Ar), krypton (Kr), xenon (Xe), radon (Rn) and oganesson (Og) and they are positioned in group 18. Among them, the last two elements are radioactive in nature. Og was only recently discovered in 2006 and its chemical and physical properties have yet to be studied in a broad sense. Very recently, because of the tremendous relativistic effect, Og was reported to possess semiconductor properties in the solid-state [2]. The main electronic characteristic of Ng atoms is their filled valence n*s*^2^ and n*p*^6^ orbitals (for He only n*s*^2^). In principle, these electronic configurations hinder these elements from getting chemically involved with other elements, which results in the isolation of Ngs in terms of reactivity and accordingly the chemistry world terms them as “inert gases”. In this group, the first ionization potentials (IP) follow a descending order along the bottom of the group (see Figure 1) [3]. If we look at these IP values, it is obvious that they possess high enough IPs to support their inertness, but another observation is that the IPs decrease monotonically from He to Rn. Thus, it is possible to knock out an electron from the outermost shell of the heavier Ngs and Ng-compounds thus become viable. Kossel predicted that Kr and Xe should be able to form the corresponding fluorides [4]. In 1924, Antropoff claimed that Ng atoms can expand their valence up to eight and should therefore be placed in Group VIIB [5]. In his words, “*one should not forget that as the valence number increases from one group to the next, the intensity of the valence forces decreases*” [6]. Pauling, based on ionic radii, predicted that Xe can coordinate with oxygen and form xenic acid (H_4_XeO_6_), which “*should form salts such as Ag_4_XeO_6_ and AgH_3_XeO_6_*” [7]. The radius ratio O^2−^ and F^−^ is 1.29, and this observation led Pauling to further predict the existence of krypton hexafluoride (KrF_6_), xenon hexafluoride (XeF_6_), and xenon octafluoride (XeF_8_). However, several attempts to synthesize Ng compounds failed until 1962. One notable mention is the electric discharge experiments to synthesize XeF_6_ or XeF_8_ by Yost and Koye. It is reported that they had tried the experiments 20 times in a week, but all the experiments failed. 

The long period of inertness of Ngs came to an end with the works of Bartlett in 1962. Bartlett accidentally synthesized the complex O_2_^+^PtF_6_^−^, which is red in color and was unexpected as per his reaction schemes [8]. The extremely high oxidizing power of PtF_6_ was able to oxidize O_2_. The IP for O_2_ to O_2_^+^ is very high (12.07 eV), which is almost equal to that of Xe to Xe^+^ (12.13 eV). Based on these similar IP values, he idealized a similar experimental procedure to make a complex between Xe and PtF_6_ and the result was positive [9]. In his own words, “*When I broke the seal between the red PtF_6_ gas and the colorless xenon gas, there was an immediate interaction, causing an orange-yellow solid to precipitate*” [10]. This ground-breaking discovery broke the century-long myth and opened a completely new field known as “Noble Gas Chemistry”. Initially, the formula of the first Ng compound was thought to be Xe^+^PtF_6_^−^, but X-ray powder diffraction photographs (XRDP) revealed the existence of XeF^+^Pt_2_F_11_^−^ but no Xe^+^PtF_6_^−^ in the solid form [11]. The mechanism of formation of XeF^+^Pt_2_F_11_^−^ was established via quantum-chemical computations by Christe [12,13]. The reaction is catalyzed by F^−^ ion and it explains the presence of XeF^+^PtF_6_^−^, PtF_5_, and XeF^+^Pt_2_F_11_^−^. 

Though Bartlett failed to give an accurate structure for the yellow solid at the time, his work opened a new branch of chemistry. The notion that Ng elements “*Do not make bond(s)*” was disproven and within a year several Ng compounds, *viz.*, XeF_2_, XeF_4_, XeF_6_, XeOF_4_, XeO_3_, and KrF_2_ were synthesized and characterized [14,15]. Xe was the most studied element among the group 18 elements due to its loosely bonded electrons and numerous reports appeared in the literature about species like XeF_2_, XeF_4_, XeF_6_, XeOF_4_, XeO_3_, and many more [16,17,18,19,20,21]. Even radioactive Rn was successfully employed to make compounds, RnF_2_ and [RnF][Sb_2_F_11_] being two examples among them [22]. In 2000, the group of Räsänen successfully isolated the first Ar compound, HArF, in a low-temperature matrix [23,24]. Several weak Ne complexes, namely NeAuF, NeBeS, NeBeCO_3_, NeBeSO_2_, (Ne)_2_Be_2_O_2_, (NeAr)Be_2_O_2_, and (NeKr)Be_2_O_2_, were also experimentally identified in a low-temperature matrix [25,26,27,28]. It should be noted that before Bartlett’s discovery, in 1925, Hogness and Lunn observed the presence of the transient species HeH^+^ by bombarding a hydrogen–helium mixture at low pressure [29]. 

**Figure 2:**
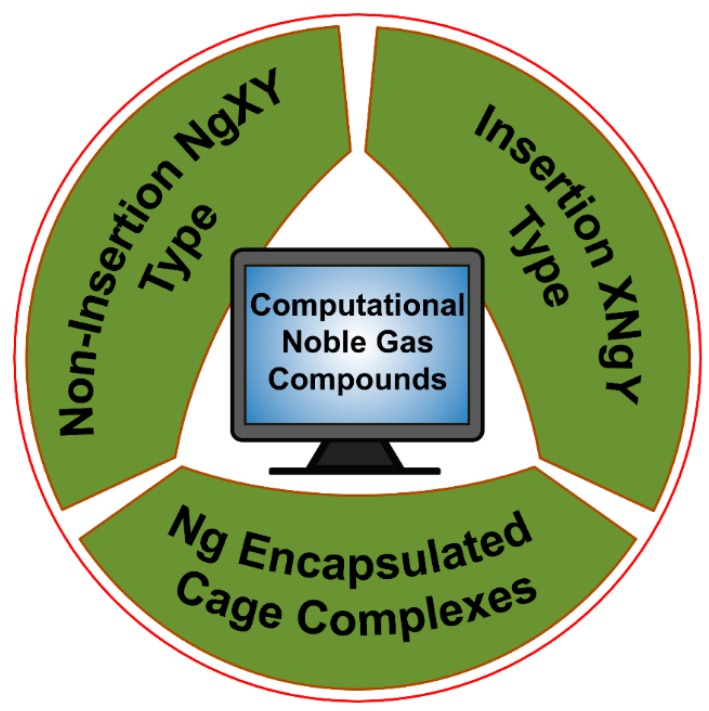
Schematic presentation of the three classes of Ng-compounds discussed in this article.

Very recently, Dong et al. showed that at high pressure even He can participate in chemical bonding [30]. They reported a solid Na_2_He compound with a fluorite-type structure under high pressures greater than 113 GPa. Bonding analysis by the solid-state adaptive natural density partitioning analysis (SSAdNDP) reveals that in the absence of He in the sodium sublattice each Na_8_ cube only has one electron. Now, in Na_2_He when helium is included in half of those cubes, the electron density from those cubes occupied by helium gets is pushed to the adjacent empty Na_8_ cube facilitating the formation of eight-centered two-electron (8*c*-2*e*) bonds therein. High pressure is needed for such electron shifting to be effective. Hence, all the Ng elements are known to show chemical reactivity. For more details, the readers are referred to the excellent reviews and articles on the Ng compounds [31,32,33,34,35,36,37,38,39,40,41,42,43,44,45,46,47,48,49,50,51,52,53,54,55]. In the current account, we mainly summarize our theoretical contributions in the Ng field. Our work on Ng compounds can be classified into three main categories, viz., (a) non-insertion of NgXY type, (b) insertion of XNgY type, and (c) Ng encapsulated cage complexes (see Figure 2).

## 2. Ng Compounds under the Light of Theoretical Chemistry

Theory complements experiments in developing and restructuring Ng chemistry. Computational tools are found to be useful in explaining the characteristics of the nature of bonding in Ng compounds. The computational results can explore that shadowy part and shed light to understand the system more conveniently, but computational chemistry is not restricted to just this application. New sets of molecules, clusters, and complexes of Ngs could be designed and their structures, stability, and nature of their bonding explored using the computational tools. 

The computational work starts with the optimization of the designed Ng compound. At this point, the primary and necessary step is to select a reliable method (*ab initio* or DFT) that predicts the geometrical, electronic, and energetic parameters with a minimum amount of errors. The *ab initio* methods like CCSD(T) along with aug-cc-pVnZ (n = T, Q, 5) or def2-nZVP (n = T, Q) basis sets are the most desirable methods for the calculations regarding the Ng compounds, but if the systems are large enough, CCSD(T) level becomes computationally expensive, and then one should go for DFT- based methods. Several benchmark studies have guided the community to find a proper DFT level. The MPW1B95/6-311+G(2df,2pd), BMK/aug-cc-pVTZ, DSD-BLYP/aug-cc-pVTZ, and B2GP-PLYP/ aug-cc-pVTZ levels provide the best accuracy for the bond energies in Ng compounds, with MUEs of ~2.0 kcal·mol^−1^ [56]. In contrast, the MP2 method along with aug-cc-pVDZ basis set gave a MUE of 4.5 kcal·mol^−1^ for these bond energies. In general, the relativistic effects of the Xe and Rn are taken care of by using either the quasi-relativistic effective core potentials (ECP) or the zeroth-order regular approximation (ZORA). Ng insertion compounds are more sensitive to the level of theory used. Very often, even if structures turn out to be a minimum at the DFT or MP2 level, at the CCSD(T) level they are shown to become dissociated. Therefore, a high-level CCSD(T) calculation is mandatory to predict new stable Ng-inserted molecules. On the other hand, NgXY-type systems are relatively less sensitive to the level of theory. Only the bond dissociation energy (BDE) changes with the change in the level of theory, but it is very unlikely that a stable species at one level turns out to be unstable at some other level.

The minimum energy geometries are then used to study the nature of bonding of the complexes. Natural bond orbital (NBO) [57], electron density [58], and energy decomposition analyses (EDA) [59,60] are performed. The NBO analysis is very widely used to find out the natural charge distribution of the molecule and to calculate the bond order between any pair of atoms by computing the Wiberg bond index (WBI). The natural charges on each atom help to determine the direction of the charge flow, whereas the WBI values indicate the bond order between two atoms. Electron density analysis helps to assign the nature of bonds based on various electron density-based descriptors. The criterion that the Laplacian of the electron density, ∇^2^*ρ*(*r_c_*), at the bond critical point (BCP) should be negative often fails for Ng compound cases, which might be because of the relatively weaker orbital involvement in Ng cases in comparison to the other elements and/or the fact that this criterion very often fails for the heavier elements other than first-row elements. This is because ∇^2^*ρ*(*r_c_*) is derived from the three curvature values (λ_1_, λ_2_, and λ_3_) where λ_1_ and λ_2_ are negative but the last term is positive. For other than first row elements the latter term very often dominates the former two terms making the resulting ∇^2^*ρ*(*r_c_*) positive. For these cases, the total energy density *H*(*r_c_*) is a very useful descriptor. Even if ∇^2^*ρ*(*r_c_*) > 0, *H*(*r_c_*) < 0 signifies covalent bonding or at least partial covalent bonding, depending on the magnitude. 

In EDA, the interaction energy (Δ*E*_int_) between two fragments is decomposed into four energy terms, *viz.*, the electrostatic interaction energy (Δ*E*_elstat_), the Pauli repulsion (Δ*E*_Pauli_), the orbital interaction energy (Δ*E*_orb_), and the dispersion interaction energy (Δ*E*_disp_). Therefore, the interaction energy (Δ*E*_int_) between two fragments can be defined as:

Δ*E*_int_ = Δ*E*_elstat_ + Δ*E*_Pauli_ + Δ*E*_orb_+ Δ*E*_disp._

Δ*E*_elstat_ is computed classically by taking the two fragments at their optimized positions but considering the charge distribution is unperturbed on each fragment, by another one. The next one is Δ*E*_Pauli_, which appears as the repulsive energy between electrons of the same spin and it is computed by employing Kohn-Sham determinant on the superimposed fragments to obey the Pauli principle by antisymmetrization and renormalization. Δ*E*_orb_ originates from the mixing of orbitals, charge transfer, and polarization between two fragments. Lastly, Δ*E*_disp_ represents the dispersion interaction between the two fragments. This method is very widely used to analyze the bonding situation in different type of systems [61,62,63,64,65,66,67,68,69,70,71].

## 3. Discussion

### 3.1. Non-Insertion Complexes of NgXY Type

In this type of Ng compounds, the Ng atoms are bound to an open side of a molecule, say X in an XY system. The X atom can polarize the electron density from the outermost orbital of the Ng atom and this produces an attractive interaction between them. The polarizing power of the atom X is responsible for the extent of the interactions between Ng and X. This interaction can be viewed as a donor-acceptor type of interaction to stabilize the Ng-X bond. Y is the counteranion that stabilizes the whole complex. It is known that X with a small radius and high charge can polarize the Ng atom more strongly. Due to the difference in electronegativities of X and Y a dipole would be created which in turn will polarize the electron cloud of the Ng atom. Of course, the strength of polarization would be much larger in case it is caused by an ion. For neutral molecules having small electronegativity differences, dispersion would also play a role in bonding. 

**Figure 3:**
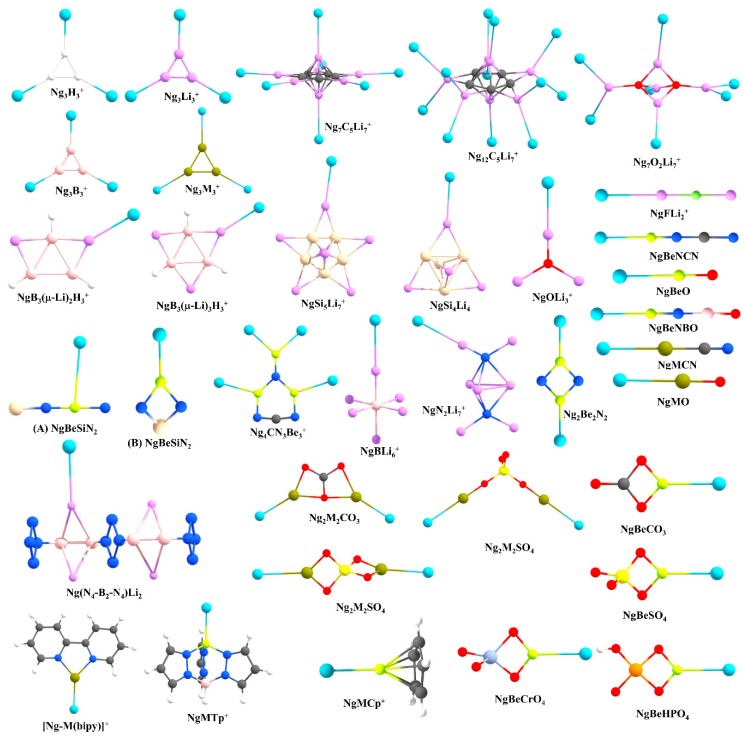
The structures of the NgXY type systems reported by us.

Prompted by the findings of Pauzat et al. [72,73,74], one of the first cases that we considered was a triangular H_3_^+^ cluster interacting with Ng atoms. Our computations showed that a maximum of three Ng atoms can form strong bonds with H_3_^+^ and the strength for the first H-Ng bond in H_3_(Ng)^+^ is significantly stronger than in larger clusters (see Figure 3 for the structures of the systems reported by us) [75]. Further, their stability could be understood in terms of conceptual DFT-based descriptors, where the overall system gets stabilized because of the presence of Ngs, presumably because of the delocalization of the cationic charge. We further compared the Ng binding ability of H_3_^+^ with Li_3_^+^. Each Li center in a Li_3_^+^ cluster can also bind with an Ng atom, resulting in a Li_3_(Ng)_3_^+^ cluster but the Ng-Li bonds are significantly weaker than the Ng-H bonds in the former systems. The Li-Ng bond strength in this system is comparable with those of the previously reported NgLiH and NgLiF systems. Note that a positive Li center is an automatic choice for hydrogen storage because of its mediocre binding ability lying between physisorption and chemisorption and high gravimetric wt% [76,77,78,79,80]. Therefore, it would be interesting to check the relative stability of H_2_ and Ng bonded analogues to understand whether a Ng can replace H_2_. Also, it is necessary to understand the origin of such an attractive interaction. We have then performed a comprehensive study on the star-shaped C_5_Li_7_^+^ cluster [81] and O_2_Li_5_^+^ super-alkali cluster [82] as a case study [83,84]. The C_5_Li_7_^+^ cluster can bind up to seven He atoms with the Li centers, whereas for other cases, a maximum of 12 Ngs (Ne-Xe) can bind. The O_2_Li_5_^+^ cluster is able to bind up to seven Ng (He-Xe) atoms. A comparison with their interaction with hydrogen molecules reveals that He and Ne have slightly lower attractive interactions than H_2_, whereas for heavier Ng atoms the interaction is stronger than that with H_2_ [78,79]. We have also considered a series of Li-decorated clusters, *viz.*, B_3_(μ-Li)_3_H_3_^+^, B_3_(μ-Li)_2_H_3_, Si_5_Li_7_^+^, Si_4_Li_4_, Ge_4_Li_4_, [N_4_-B_2_-N_4_]Li_2_ and super-alkali clusters (FLi_2_^+^, OLi_3_^+^, N_2_Li_7_^+^, and BLi_6_^+^) to justify that such bonding with Ng is not specific to certain Li-decorated clusters but, in general, a cluster with considerably positively charged Li center can induce a bonding with Ng [83,85]. Moreover, the application of an external electric field improves the Li-Ng bonding. EDA reveals that charge transfer and polarization are the main contributors towards the total attraction, followed by exchange and dispersion interactions.

The elements of group 2, mainly beryllium, have shown their potential in this field. Be has significantly larger Ng binding ability than Li because of its higher ionic potential. In 1988, Frenking and co-workers showed on paper that BeO can form strong bonds with Ng atoms in NgBeO complexes [86]. Later, in 1994, Andrews and co-workers detected ArBeO, KrBeO, and XeBeO complexes using the pulsed-laser matrix isolation technique [87]. Moreover, the applied charge on BeO increases the Ng-Be bond strength [88]. These findings motivated us to search for viable Ng-Be complexes. A global minima search for CN_3_Be_3_^+^ molecular formula shows that it has *C*_2*v*_ symmetry, where two different types of Be centers are present (see Figure 3) [89]. Each Be center connected through the ring can bind only one Ng atom, but the Be center outside the ring can bind two Ng atoms, thus making a total of four Ng-atoms bonded to the system. The thermochemical results indicate that the NgCN_3_Be_3_^+^ cluster might be viable even at ambient temperature, whereas for higher-numbered Ng bound clusters, the temperature should be lowered. Quite high WBI values reveal that almost half a bond order is attained for Ng = Kr-Rn. In the same study, the whole series of NgBeX (X = O, S, Se, Te) and their cationic analogues were also considered. For a given X, the cationic species have higher Ng binding ability than their neutral analogues, whereas for a given Ng, the Ng-Be strength diminishes along O to Te. In a further study, we searched for the global minimum structures of Be_2_N_2_, Be_3_N_2_, and BeSiN_2_ clusters [90]. Owing to a high positive charge, the Be-center in these clusters can bind to Ng atoms. Be_2_N_2_, Be_3_N_2_, and BeSiN_2_ bind a maximum of two, three, and one Ng atoms through the Be centers, respectively. An interesting finding is that the presence of Ng atom can alter the energetic sequence of the isomers through their interaction with them. For example, the global minimum structure of BeSiN_2_ has a linear shape (**A** in Figure 3), where Be is located in between two atoms, and therefore, Be is not in a suitable position to interact strongly with Ng atoms (Figure 3). On the other hand, in the second lowest-lying isomer the Be is in a terminal position with high positive charge and can interact Ng atoms strongly (**B** in Figure 3). It was noted that in the presence of Ar-Rn, the second-lowest energy isomer becomes more energetically stable than the linear global minimum isomer. These are important results since many clusters are generated experimentally in a Ng (Ar) environment! 

Various ways to modify the Ng binding strength of BeO and BeNH were investigated thoroughly. For example, the attachment of a Lewis acid like BH_3_ to the O atom of BeO was found to improve the Ng binding ability as reported by Grandinetti and co-workers. The same authors also noted that the substitution of H atom in BeNH by different groups makes it a better candidate to bind Ng atoms [91], but the major drawback of this approach is that the designed systems are not global minima. We found two Be-containing molecules, BeNCN and BeNBO, via substitution of the H atom of BeNH with CN and BO, respectively, which have a linear global minima structures with Be in a terminal position [92]. These two systems have the highest and the second-highest Ng binding ability among other reported neutral Be systems. Further, the quest to find suitable Be-based salts that can form strong Ng-Be bonds led to the study of the Ng binding ability of several BeX (X = SO_4_, CO_3_, HPO_4_, and CrO_4_) systems [93,94]. The comparative study on the NgBeSO_4_, NgBeCO_3_, and NgBeO clusters suggests that the Ng-Be bonds are stronger in NgBeSO_4_ as compared to those in NgBeCO_3_ and NgBeO (except for the He-Be case) clusters. 

**Figure 4:**
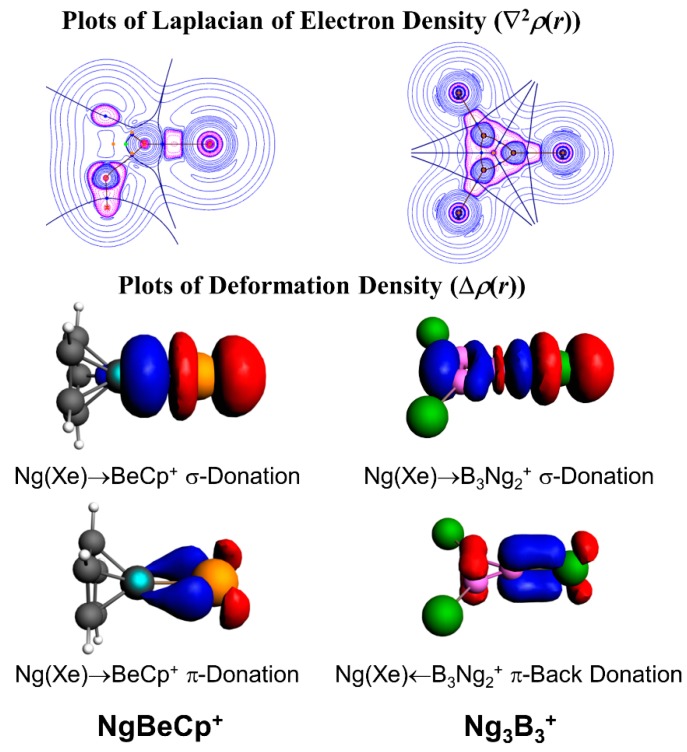
The contour plots of Laplacian of electron density in Ng_3_B_3_^+^ complex (pink for negative values and blue for positive values) and deformation density plots in NgBeCp^+^ and Ng_3_B_3_^+^. Electron density moves from red to blue region. This figure is reprinted with permission from [96], copyright 2017 American Chemical Society. Part of the figure is also reproduced from [97] with permission with permission from The Royal Society of Chemistry.

In a further subsequent study, the Ng binding ability of Be and Mg salts of 1-tris(pyrazolyl)-borate was studied by DFT-based studies [95]. The Ng-Be bonds are stronger than that of the Ng-Mg bonds, which is the direct consequence of the small charge to radii ratio in Mg as compared to that in the Be. In these positively charged complexes, the M centers polarize the electron density on the Ng atoms. The EDA in combination with natural orbital for chemical valence (NOCV) also showed that the orbital interactions are the major contributing factors here. In another case, we reported the Ng gas bound half-sandwich complexes, NgMCp^+^ (M = Be-Ba; Ng = He-Rn, Cp = *η*^5^-cyclopentadienyl anion) [96]. In these cases, the dissociation energy values follow the order as Ng-Be > Ng-Mg > Ng-Ca > Ng-Sr > Ng-Ba for a particular Ng atom, whereas for an M atom, the dissociation energy gradually increases along He-Rn. Figure 4 displays the deformation densities associated with the two major orbital contributors. In the plot, the electron density is shifted from red to blue. The major orbital contribution is originated from the Ng(p_σ_)→BeCp^+^ σ-donation, whereas Ng(p_π_)→BeCp^+^ π-donation is responsible for the next orbital term. The plot of ∇^2^*ρ*(*r*) also shows electron density accumulated region (pink color) in between Ng and Be, indicating the covalent bond formation.

We also focused on the next neighbor of Be, boron, which is inherently electron deficient in nature, and therefore, is expected to act as strong electron acceptor from Ng. The B_3_^+^ cluster is a π-aromatic system [97]. We tested the Ng binding ability of this B_3_^+^ cluster for He-Rn which leads to the formation of a B_3_(Ng)_3_^+^ cluster. The He-B and Ne-B bonds are very weak, but for the heavier Ng atoms, the Ng-B (Ar-Rn) bonds are significantly strong (ranging 7.0–13.1, 14.0–32.0, and 24.4–53.2 kcal·mol^−1^ for the third, second, and first Ng-B bonds, respectively). The strong interaction is also reflected from the corresponding WBI values of 0.57–0.95. Further, the contour plot of ∇^2^*ρ*(*r*), and EDA-NOCV results show that the Ng-B (Ar-Rn) bonds can be designated as covalent (Figure 4). Later, another computational work by Li and co-workers revealed that B_n_^(n−2)+^ clusters could be able to bind with nNg atoms and the results corroborate our findings [98,99,100].

Next, we examined the Ng-binding ability of group 14 elements. The cationic EX_3_^+^ (E = group 14 elements, X = H, F, Cl, Br) were also found to have strong Ng binding ability, albeit lower than the boron centers in B_3_^+^ [101,102]. For a given Ng, EH_3_^+^ follows the Ng binding order C > Si > Ge. For Sn and Pb cases, *D*_3h_ symmetric EH_3_^+^ is not a global minimum, rather EH···H_2_^+^ is the most viable one. In the replacement of -H by -X (X = F, Cl, Br), two opposing effects, viz., the -I (inductive) effect of X would enhance the Lewis acidity of E and X→E π-back-donation would diminish its Ng binding ability. The overall situation would depend on the balance between these two factors. EF_3_^+^ (E = Si, Ge) was noted to have increased Ng binding ability compared to the corresponding EH_3_^+^. However, because of the very efficient F→C π-back-donation in CF_3_^+^, it has significantly lower ability to interact with Ng in comparison to CH_3_^+^. It is also shown that the EH_3_^+^ (E = Si, Ge) and EF_3_^+^ (E = Si-Pb), can bind with two Ngs effectively at the same time. The large Lewis acidity for this group of systems was also found for the Lewis base CO [103].

Apart from these main group elements, we have also studied the possibility of transition metal compounds bonding with Ng. A series of superhalogen molecules with general formula MF_3_ (M = Ru, Os, Rh, Ir, Pd, Pt, Ag, Au) was considered and their Xe binding ability was tested. Among this series, in terms of bond dissociation energy, RuF_3_ and AuF_3_ are the best and second best candidates to bind Xe [104]. Thereafter, in a series of studies, we found that the complete series, Cu, Ag, and Au, exhibits remarkable Ng binding ability, which is, in general, larger than the other transition metals. At first, we considered the σ-aromatic triangular M_3_^+^ (M = Cu, Ag, Au) clusters and the possibility of forming M_3_(Ng)_3_^+^ complexes [105]. The Ng-M bond dissociation energies that range within 2.2–19.0 kcal·mol^−1^ follow a descending order of Au > Cu > Ag, for a given Ng atom. In contrast to the main group elements, where the orbital contribution is found to be the dominant term, the Ng-M bond for these cases is supported by both Coulombic and covalent contributions in almost equal strength. In the orbital term, the major contributing factor that stabilizes the Ng-M bonds is the electron donation from one of the filled *p*-orbitals of Ng-atoms to the LUMO of M_3_^+^ clusters. In subsequent studies, rather strong ‘noble-noble’ interaction was understood through the analysis of NgMNO_3_, NgCu(NO_3_)_2_, NgMSO_4_, Ng_2_M_2_SO_4_, NgCuCO_3_, Ng_2_M_2_CO_3_, NgMCN, NgMO, and monocationic Ng bound M-bipyridine [Ng-M(bipy]^+^ complexes (Ng = Ar-Rn; M = Cu, Ag, Au) [106,107,108,109,110]. A partial covalent character of the Ng-M bonds (Ng = Ar-Rn) was understood via the electron density and EDA-NOCV analyses. For [Ng-M(bipy]^+^, a major demand to synthesize these cationic complexes is to find a suitable counterion that can stabilize the whole complex without weakening the Ng-M bonds. The [SbF_6_]^−^ anion was noted to serve this purpose well. For further information about the advancement in the field of noble metal noble gas chemistry, readers are referred to a recent review [111].

### 3.2. Insertion Complexes of XNgY Type

**Figure 5:**
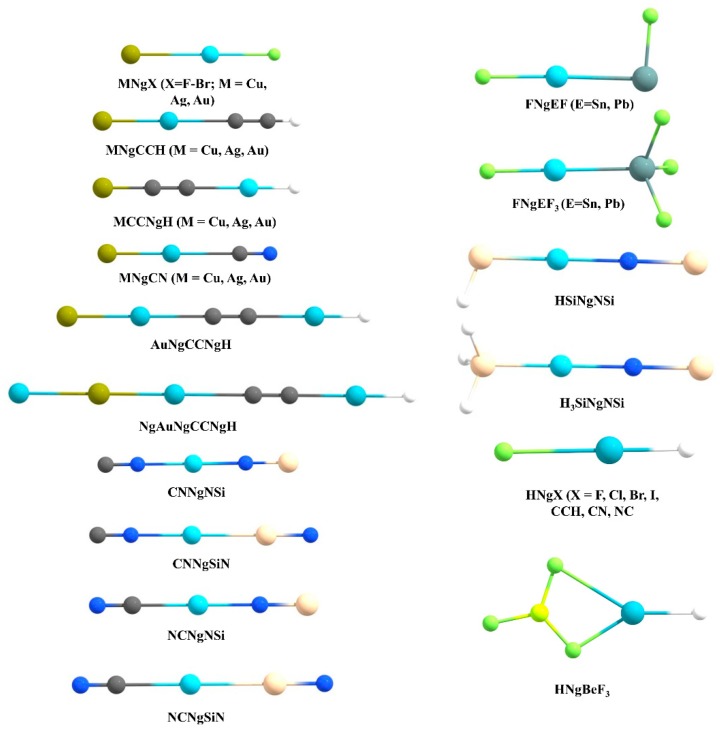
The structures of the XNgY type systems reported by us.

Another widely studied class of Ng compounds are the XNgY type where the Ng is inserted within an X-Y bond (see Figure 5 for the systems reported by us). So far, in literature a good number of Kr and Xe inserted compounds were isolated in low-temperature matrices [34,36,40], while HArF is the only example for Ar insertion [23]. There is still no experimental report of He or Ne insertion compounds.

**Figure 6:**
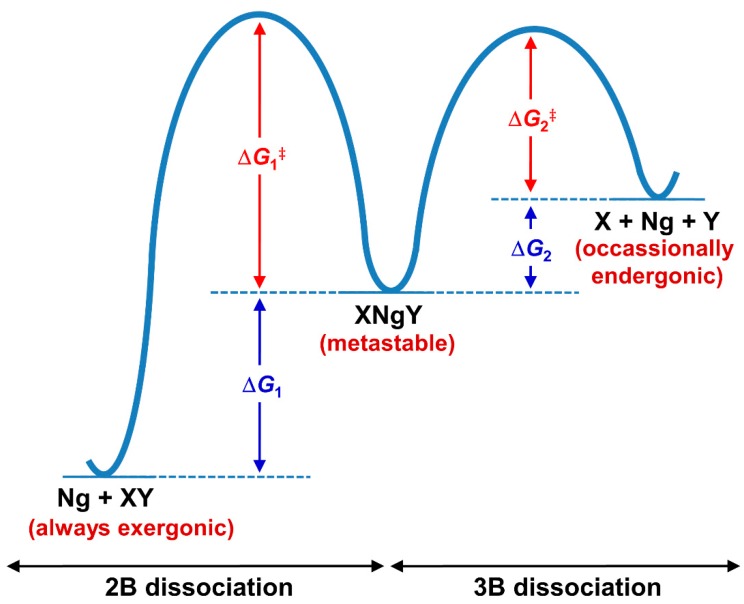
The schematic representation of the 2B and 3B dissociation paths of XNgY.

In comparison to the NgXY type, there are some basic differences in the stability of this category. The stability in the former is dictated by the thermodynamic norms of Ng-X bonds. A strong polarizing center always shows strong donor-acceptor interaction with Ng atoms. On the other hand, the stability of XNgY type of compounds is not straightforward as the formation of such molecules needs to compromise the X-Y interaction. Because of the lower reactivity of Ng, in no cases can the sum of X-Ng and Ng-Y interactions compensate the X-Y interaction. Therefore, such molecules are not thermochemically stable species, and rather their existence is driven by kinetics. Generally, for these cases, a large number of dissociation paths need to be considered. The thermochemical studies show that except for the two dissociation channels (shown below), others are highly (or at least moderately) endergonic in nature showing the stability of XNgY. The two most competing dissociation paths are: (i) two-body (2B) dissociation path: XNgY → Ng + XY and (ii) three-body (3B) dissociation path: XNgY → X + Ng + Y. In general, the 2B dissociation channel is highly exergonic in nature, whereas the 3B dissociation occasionally becomes slightly exergonic at room temperature. Therefore, to comment on the stability of XNgY, one should check the activation energy barrier for these paths (see Figure 6). While in most of the cases single reference-based methods are quite reliable to study the transition state (TS) for 2B dissociation, the computation for the barrier of 3B dissociation is complicated as simultaneous bond-breaking that often requires multireference treatment, and, therefore it is hard to evaluate for slightly larger systems. Best systems are those for which the 3-B dissociation path becomes endergonic in nature, at least at low temperatures. Hu and co-workers [112] showed by computations that the half-life of XNgY type of Ng compounds depends on the energy barrier height. An XNgY system with a minimum energy barrier of 6, 13, and 21 kcal·mol^−1^ would have a half-life in the order of ~10^2^ s at 100, 200 and 300 K, respectively. 

**Figure 7:**
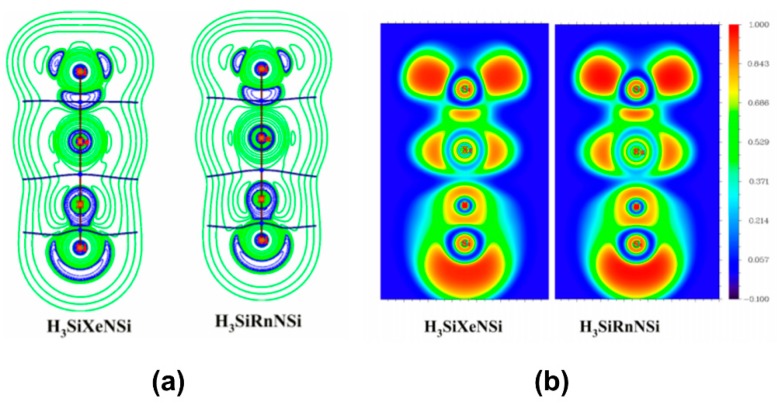
Contour plots of (**a**) Laplacian of electron density and (**b**) electron localization function of H_3_SiNgNSi (Ng = Xe, Rn).

In a couple of studies, Merino and co-workers [113,114] studied the bonding situation of HNgY (Y = F, Cl, Br, I, CCH, CN, NC; Ng = Xe, Rn) molecules where the H-Ng bonds were found to be electron-shared bond and Ng-Y was an ionic bond. Overall, the molecules might be considered as the interaction between Ng^+^ and [H^…^Y]^−^ that form a polar electron-shared bond. Both the molecules are metastable in nature having sizable kinetic protection against dissociation: HNgY → Ng + HY. Thereafter, some of us showed that H_3_SiNSi and HSiNSi molecules can also form Ng insertion compounds like H_3_SiNgNSi and HSiNgNSi (Ng = Xe, Rn), respectively [115]. The free energy change (Δ*G*; computed at 298 K and 1 atm) associated with the 2B dissociation channel producing the free Ng and parent molecule is negative in both the cases. On the other hand, the 3B dissociation path is endergonic for the Rn analogues but slightly exergonic (small negative Δ*G*) in the case of Xe, though computations at lower temperatures make this process endergonic. Negative ∇^2^*ρ*(*r_c_*) and *H*(*r_c_*) values and high electron localization function (ELF) at Si-Ng BCP reflect the covalent nature of bonding, whereas Ng-N bond is of ionic type (see Figure 7). In fact, the systems could be best represented as (H_3_SiNg)^+^(NSi)^−^ and (HSiNg)^+^(NSi)^−^ as revealed by NBO analysis. EDA also corroborates this argument where the Si-Ng bond is dominantly supported by Δ*E*_orb_, whereas in the Ng-N bond the Δ*E*_elstat_ contribution is substantial. The computed activation energy barriers suggest that the H_3_SiNgNSi is kinetically stable enough to be detected at the 250–300 K temperature range, whereas HSiNgNSi needs a lower temperature range (150–200 K) to be detected.

We extended our work in this category of Ng compounds with the report of the first set of compounds having E-Ng (E = Sn, Pb) covalent bonds, particularly FNgEF_3_ and FNgEF (E = Sn, Pb; Ng = Kr, Xe, Rn) molecules [116]. They are found to be metastable in nature, where only FNgEF_3_ → Ng + EF_4_ and FNgEF→ Ng + EF_2_ are highly exergonic, but they are protected by an energy barrier of 23.9–49.9 kcal·mol^−1^ for the former case and 2.2–8.7 kcal·mol^−1^ in the latter system, with a gradual increase from Kr to Rn. The natural charge distribution, corresponding *H*(*r_c_*) values, and the EDA computations conclude the covalent character in Ng-E bonds and an ionic description in Ng-F bonds. 

A recent work by Samanta reported superhalogen (BO_2_, BF_4_)-supported Ng insertion compounds, HNgY (Y = BO_2_, BF_4_) which are more stable than their halogen analogues [117]. Therefore, a superhalogen is a more efficient candidate than a halogen to form Ng insertion compounds. As reflected above, the Be center has an excellent capability to form viable non-insertion type regular Ng compounds which prompts us to study the performance of the Be-based superhalogen BeF_3_ in stabilizing the HNgBeF_3_ (Ng = Ar-Rn) compounds [118]. Between the 2B (HNgBeF_3_ → Ng + HBeF_3_) and 3B (HNgBeF_3_ → H + Ng + BeF_3_) dissociation paths, 2B dissociation one is exergonic whereas the 3B dissociation option is endergonic. Another pathway (HNgBeF_3_ → Ng + HF + BeF_2_) is also exergonic, but careful observation leads us that this is not a single step process rather than two as, HNgBeF_3_→ Ng + (HBeF_3_) → Ng + (HF + BeF_2_). The barriers for the 2B dissociation processes are 1.0–13.9 kcal·mol^−1^ for Ar to Rn compounds. The Xe and Rn analogues might be stabilized through a half-life of 10^2^ s up to 100 K temperature. Thorough bonding analysis shows that the H-Ng bonds are of covalent type, whereas the Ng-F interactions are of ionic-type and the molecule could be best represented as (HNg)^+^(BeF_3_)^−^.

**Figure 8:**
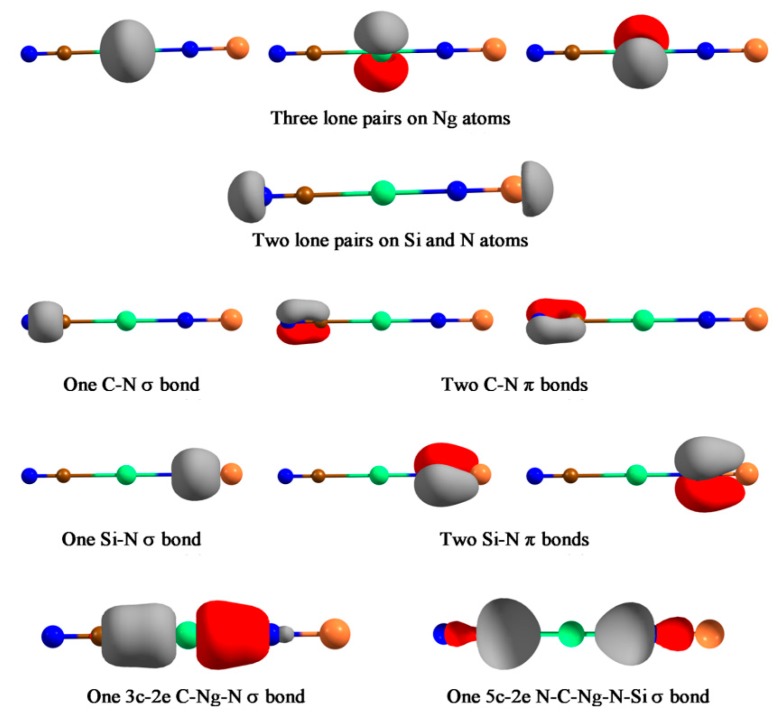
The bonding elements recovered by the AdNDP analysis for NCNgNSi (Ng=Kr–Rn) compounds. The occupation numbers in all cases are very closed to 2.00. This figure is reproduced from [119].

Next, we reported a special system, NCNgNSi (Ng = Kr, Xe, Rn), which represents the first case of a covalently bound C-Ng-N unit [119]. XNgY insertion molecules are, in general, represented as X^+^(NgY)^−^. However, these molecules feature the unprecedented case where both C-Ng and Ng-N bond can be represented by an electron-shared bond. Except for a 2B dissociation path, NCNgNSi → Ng + CNSiN, all other dissociation channels are endergonic for Xe and Rn analogues. For NCKrNSi the 3B dissociation channel producing CN, Kr, and NSi, is slightly exergonic at 298 K, thus a lower temperature will make this dissociation channel endergonic. The free energy barrier for the 2B dissociation ranges from 25.2–39.3 kcal·mol^−1^ for Kr to Rn analogues, with a gradual increase along Kr-Rn is obtained. Thus, the NCNgNSi (Ng = Xe, Rn) systems are viable and may be detected under ambient conditions, whereas the Kr analogue would be detectable at low temperature. The simultaneous existence of C-Ng and Ng-N covalent bonds is confirmed from the sizable WBI value (> 0.5), negative *H*(*r_c_*) values, and the EDA computations. In the latter analysis, the size of the Δ*E*_orb_ term is a very useful indicator to justify which fragmentation scheme is the best to represent the electronic structure of the overall molecule. The one, which results in the lowest Δ*E*_orb_ value, is the best to describe the bonding of the molecule as it needs the least change in charge distribution to get the electronic structure of the molecule [120,121]. For both C-Ng and Ng-N bonds, we tested both neutral (electron-shared) and ionic schemes, and in either case the neutral scheme results in the lowest Δ*E*_orb_ value. AdNDP analysis further corroborates this description which identifies a delocalized 3*c*-2*e* σ-bond within the C-Ng-N moiety and a totally delocalized 5*c*-2*e* σ-bond in these molecules (see Figure 8). Another interesting fact is that the dissociation of Ng from NCNgNSi produces a higher energy isomer, CNSiN, rather than the most stable parent moiety NCNSi. As such the NCNSi → CNSiN process needs to cross a very high barrier, but in the presence of Ng it gets reduced substantially, hence, Ng could facilitate the detection of the high energy isomer of NCNSi. 

**Figure 9:**
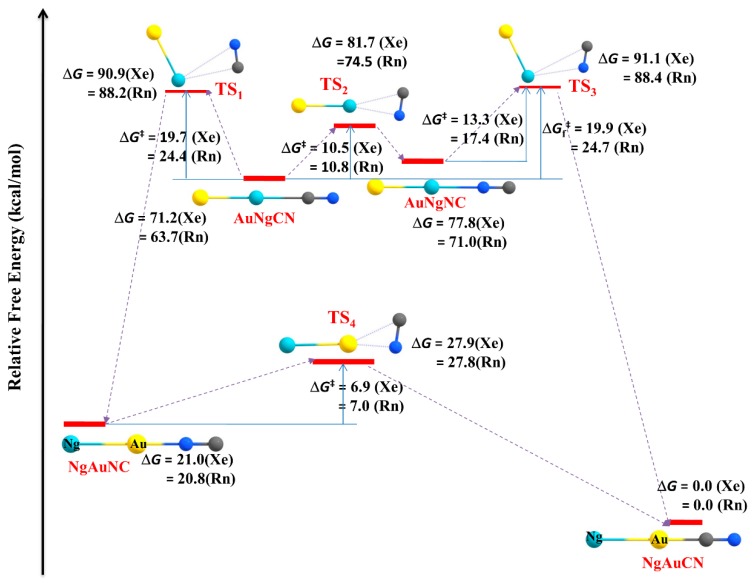
The isomeric transformation occurred in AuNgCN molecules to be converted into the most stable NgAuCN form studied at the MPW1B95/cc-pVTZ/cc-pVTZ-PP level. Relative free energies with respect to the most stable NgAuCN isomer in kcal·mol^−1^. This figure is reproduced from [123] with permission from the Centre National de la Recherche Scientifique (CNRS) and The Royal Society of Chemistry.

Apart from the main group elements, we have also extended our work to transition metal systems. Transition metal acetylides (MCCH; M = Cu, Ag, Au) are excellent in forming Ng insertion- type compounds. Ng atoms can be inserted into C-H and/or M-C bonds in the MCCH molecules [122,123]. The MNgCCH (Ng = Xe, Rn) [123] complexes are the first set of examples having M-Ng-C motif in the chemical literature. The isomerization process, MNgCCH → NgMCCH, i.e., insertion to non-insertion type, is exergonic in nature. Except for the Ag analogues, the 3B dissociation channels are endergonic in nature. The computed barrier shows that these complexes are indeed viable. The M-Ng and Ng-C bonds are covalent and ionic in nature, respectively. The best representation of the complexes is (MNg)^+^(CCH)^−^. Another possible position for Ng insertion in MCCH is within C-H bondz which results in the formation of MCCNgH [122]. Here, the bonding analysis indicates that the H-Ng bond is covalent in nature whereas the Ng-C bond has a partially covalent character. Furthermore, two Xe inserted analogues, AuXeCCXeH complex and the three Xe bond system, XeAuXeCCXeH, might also be viably detected in a low-temperature matrix. In a subsequent study, the possibility of Ng insertion into the M-C bonds of metal cyanides (MCN) resulting in the compounds with the formula, MNgCN (M = Cu, Ag, Au; Ng = Xe, Rn) was carried out [124]. The compounds MNgCN are thermochemically stable with respect to all possible dissociation paths, except for two 2B dissociation processes, *viz.*, MNgCN → Ng + MCN and MNgCN → Ng + MNC. These two dissociation paths can be further connected with the internal rotation of [MNg ↔ NgM] and [CN ↔ NC] as in, MNgCN → NgMCN and MNgCN → NgMNC, respectively. These processes are kinetically protected by substantial nergy barriers (11.8–15.4 kcal·mol^−1^ for Cu, 9.8–13.6 kcal·mol^−1^ for Ag, and 19.7–24.7 kcal·mol^−1^ for Au). The former process occurs in two steps via an intermediate MNgNC, whereas the latter one is a single step process. NgMNC can isomerize into a more stable NgMCN, but a certain energy barrier protects it. The process involves two steps for Cu but single step for Ag and Au analogues, and most importantly corresponding barrier enhances in comparison to that in bare MNC→MCN process (see Figure 9). 

### 3.3. Ng-Encapsulated Cage Complexes 

The effects of confinement on quantum-chemical entities change their physical and chemical properties up to a certain extent. Special restrictions applied since the confinement changes the electronic energy levels, linear and non-linear optical properties, nature of bonding, the selectivity of chemical reactions, etc. The particle-in-a-box is the long-known pedagogical problem that every chemistry student learns. A real-life observation is the formation of *endo* product between the reaction of 9-hydroxymethylanthrancene and N-cyclohexylphthalimide in presence of Pd(II) self-assembled coordination cages [125].

**Figure 10:**
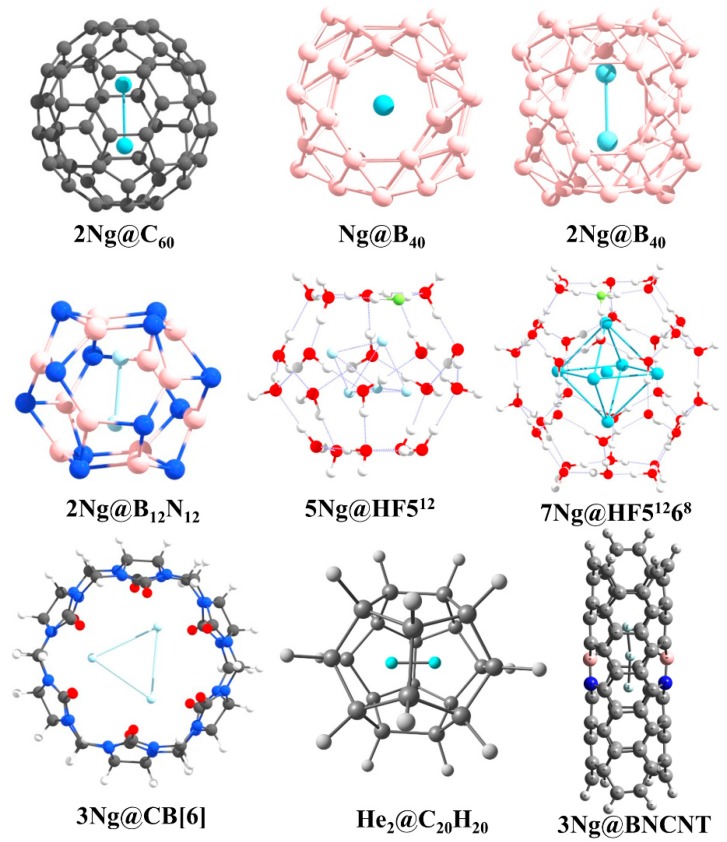
The structures of the Ng-encapsulated cage systems reported by us.

Confinement affects the bonding between two Ng-atoms. Fullerenes are one of the most studied cavitands that have been used to study the effects of confinement. Particularly, C_60_ is used to trap two Ng atoms in Ng_2_@C_60_ (Ng = He, Ne, Ar, Kr) [126,127] (see Figure 10 for the structures of all Ng- encapsulated cage complexes reported by us). Krapp and Frenking [126] showed that because of confinement within C_60_, the bond distance in Xe_2_ even becomes smaller than that in free Xe_2_^2+^. Bonding analysis reveals that because of strong steric pressure exerted by the host, two Ng atoms come closer to each other which results in a strong enough orbital involvement to give negative *H*(*r*_c_) values at the BCP of Ng-Ng bond for Ng = Ar, Kr, Xe with gradually larger negative values in going from Ar to Xe. By comparing the reactivity of the encapsulated Ng_2_ with the free one, they concluded that the Ng-Ng bond in Ng_2_@C_60_ should be called as a true chemical bond for Ng = Ar-Xe, whereas in cases of Ng = He and Ne, because of lower sizes the cavity space turns out to be large enough to orient in some distant position in order to minimize the Pauli repulsion between each other and interacting through van der Waals interaction. We complemented this study through our ab initio molecular dynamics study to examine the kinetic stability of Ng_2_@C_60_ [127]. During our simulation, we perceived the precessional movement of Ng_2_ inside the C_60_ cage where Ng_2_ acts as a single chemical entity which means that no haphazard movement is noted. The movement of two Ng atoms correlates with each other. This result also supports the formation of some sort of bonding between two Ng atoms after the confinement. With the increase in the size of the Ng atom, the extent of such precessional movement diminishes which is because of the facts that with the increasing size of the heavier Ng atoms, the interaction between that Ng and carbon atoms of the cage wall also increases and with the larger sizes the reorganization energy of the cage for the movement of Ng_2_ also increases. In another relevant study, we found that the encapsulation of Xe_2_ dimer demands so significant structural change in the host C_60_ moiety that the latter breaks the isolated pentagonal rule (IPR) [128], rather it prefers a geometry having two adjacent pentalene units in order to provide larger space to Xe_2_ [129].

Then, some of us attempted to even induce a chemical bond between two He atoms by confining in a significantly smaller cavity than C_60_ [130]. Because of the confinement of two He atoms within C_20_H_20_ dodecahedrane, the shortest He-He internuclear distance of 1.265 Å is attained which is even less than half of the distance in free He_2_ dimer. Although a very short internuclear distance and a bond path is obtained between two He atoms, negligible charge transfer, essentially zero WBI value and the corresponding plots of ∇^2^*ρ*(*r*) and molecular orbitals (MOs) suggest a closed-shell interaction between them. Based on this study, the drawn conclusion is that *a short internuclear separation does not necessarily imply the existence of a chemical bond.* This conclusion also corroborates the argument that a bond path does not necessarily imply a chemical bond [131].

We continued our effort to get a chemically bound He_2_ unit under a confined situation. We have now tested within B_12_N_12_ and B_16_N_16_ cages [132]. Although such encapsulated analogues are thermochemically unstable with respect to dissociation, they are kinetically stable as understood from the ab initio simulations. In these heteroatomic cages, the charge transfer from He to the cage slightly improves in comparison to that in C_20_H_20_. Interestingly, in case of He_2_@B_12_N_12_ the *H*(*r*_c_) values at the BCP of He-He bond turns out to be negative (−0.009 au), indicating some sort of orbital (covalent) involvement therein. Further, EDA computation taking two He atoms as two fragments with a frozen distance as in the encapsulated complex reveals that 40.9% of the total attraction is originated from the Δ*E*_orb_ term which corroborates with the orbital involvement. 

The clathrate hydrate and its HF doped analogue can also encapsulate Ng atoms [133]. A model study considering 5^12^, HF5^12^ (the HF doped analogues), 5^12^6^8^ and HF5^12^6^8^ as host cages and Ng = He, Ne, and Ar as target species has been performed thoroughly. The HF doping increases the stability of the cages as well as that of Ng encapsulated cages. The 5^12^ and HF5^12^ cages encapsulate one Ng atom in all cases. The HF5^12^6^8^ cages can hold up to 10 He, 6 Ne atoms and 6 Ar atoms depending on the temperatures. The Ng-O, Ng-F, and Ng-Ng interactions in these cases are found to be purely noncovalent in nature. 

We further extended our work to the cucurbit[n]uril (CB[n]) taking CB[6] as a case study. CB[n] is well-known for its host-guest chemistry [134,135,136]. We employed them to encapsulate Ng atoms as well [137]. CB[6] could encapsulate up to three Ne atoms whereas two Ng (Ar and Kr) inside the cavity. The larger sizes of Ar and Kr as compared to smaller Ne are responsible for this. The repulsive force between the Ngs and CB[6] distorts the cage walls. The distances between two Ng atoms are smaller than the summation of their van der Waal’s (vdW) radii. The electron density analysis shows that the interactions between the Ng atoms inside the CB[6] cage is closed-shell type in nature. The dispersion term is the major contributing one that stabilizes the whole system as revealed by EDA. The molecular dynamics simulations show that at 77K the Ng atoms would remain inside the cage. A similar study was also carried out with octa acid [138].

BN-doped carbon nanotubes (BNCNTs) have shown that not only the confinement but also the electronic charge distribution of the cavitand plays an important role in stabilizing Ng atoms inside the cavity [139]. The He-He bond distance is optimized to be 1.824 Å in He_2_@BNCNT, which is surprisingly smaller than that in He_2_@CNT (2.596 Å; CNT denotes the pristine carbon nanotube). The induced polarization of the He atoms is more in case of He_2_@BNCNT as compared to the He_2_@CNT. A similar type of result is also true for the Ng-Ng distances in Ng_n_@BNCNT (Ng = He (n = 3), Ar (n = 2), Kr (n = 2)). The closed-shell interaction of the He-He and partial covalent characters of Ar-Ar and Kr-Kr inside BNCNT cavity are revealed by AIM analysis. 

Borospherene (B_40_) can also encapsulate Ng atoms and systems like Ng_n_@B_40_ (n = 1 for He-Rn; n = 2 for He-Kr) [140] have been studied using DFT methods. The systems are not thermodynamically stable with respect to the dissociation to B_40_ cage and free Ng atoms, but once the entrapped cages are formed, they are kinetically stable due to a high free energy barrier (Δ*G*^‡^). The Δ*G*^‡^ values range from 84.7–206.3 kcal·mol^−1^ for Ng@B_40_ (Ng = He-Rn) systems. The increase in the size of the Ng atom increases the distortion in the encapsulated B_40_ cage in each case. This size effect is enough to explain optimization of Ng_2_ dimers up to Kr analogues inside the B_40_ cage. The Ar-Ar and Kr-Kr bonds acquire partially covalent character upon encapsulation. The encapsulation of Ng-atoms changes the fluxional behavior and reactivity of B_40_ cages. B_40_ cage exhibits fluxionality [141,142,143,144,145] which includes a continuous transformation between the B_7_ and B_6_ holes [146]. The Δ*G*^‡^ for this transformation increases upon encapsulation of Ng atom. Furthermore, the presence of Xe inside the B_40_ cage enhances the complexation ability of B_40_ with [Fe(*η*^5^-C_5_Me_5_)]^+^ as compared to the cage without Xe atom. Therefore, the above works show that the confined systems may exhibit different and unusual behavior from their free states which has been further highlighted in a recent perspective article [147]. 

## 4. Conclusions

The present account summarizes our contributions to predicting new Ng compounds and the bonding therein. We have worked on three different types of Ng complexes, *viz.*, non-insertion compounds of NgXY type, insertion compounds of XNgY type and Ng encapsulated cage complexes, where X and Y represent any atom or group of atoms. Depending on the strength of the interaction, NgXY might be thermochemically stable at a certain temperature. However, the other two categories are kinetically stable since XNgY always possesses an exergonic dissociation channel, XNgY → Ng + XY, and because of the limited space the presence of Ng causes steric crowding in Ng encapsulated cage complexes. Therefore, their stability would rely on the activation energy barrier. XNgY molecules are very sensitive to the level of theory. Often a system which is a minimum at DFT level, may dissociate during optimization at the CCSD(T) level. Therefore, a study at the CCSD(T) level for this category is highly recommended. For the other types, the bond dissociation energy may vary with the change in the level of theory but such a drastic change in the stability was not found. The bonding analysis is thoroughly scrutinized by employing full theoretical arsenal, *viz.*, NBO, electron density analysis and EDA. Occasionally, they are coupled with AdNDP analysis. In NgXY, the strength of donor-acceptor Ng→XY interaction depends on the polarizing power of binding X center to draw the rather rigid electron density of Ng towards itself, and sometimes involvement of such orbitals becomes large enough, particularly for heavier Ng elements, to consider them as covalent bonds. On the other hand, in most of the XNgY cases Ng forms electron-shared covalent bonds with X while interacting electrostatically with Y, thus representing itself as [XNg]^+^Y^−^. Nevertheless, in some of the rare cases like NCNgNSi, both the C-Ng and Ng-N bonds can be represented as electron-shared covalent bonds. On the other hand, a cage host is an excellent moiety to examine the limits that can be pushed to attain bonding between two Ng atoms (even for He) at high pressure. The confinement effect by a small cage-like B_12_N_12_ can even induce some covalent interactions between two He atoms in He_2_@B_12_N_12_. Starting very lately, the chemistry of noble gases is now at its young age and is developing very rapidly. We believe that Ng has very widespread hidden chemistry, just waiting to emerge soon!
